# Medical students’ self-evaluation of character, and method of character education

**DOI:** 10.1186/s12909-022-03342-6

**Published:** 2022-04-13

**Authors:** Yera Hur, Sanghee Yeo, Keumho Lee

**Affiliations:** 1 Institute of Medical Education, College of Medicine, Hallym University, Chuncheon, Republic of Korea; 2 Center for Medical Education, School of Medicine, Kyungpook National University, Kyungpook National University Hospital, Daegu, Republic of Korea; 3 Center for Liberal Arts, Korea University of Technology & Education, Cheonan, Republic of Korea

**Keywords:** Character, Medical students, Professional practice gaps, Self-assessment, Professionalism

## Abstract

**Background:**

As medical doctors must have a strong sense of ethics, character education is particularly important for them compared with other professions. This follow-up study aimed to establish the foundation for developing a character education program in medical schools by (1) conducting a survey among medical students on the self-assessment of one’s character based on eight qualities (service and sacrifice, empathy and communication, care and respect, honesty and humility, responsibility and calling, collaboration and magnanimity, creativity and positivity, patience and leadership), the perceived importance of character, and satisfaction with character education at medical school, and (2) analyzing the usefulness of learning methods for acquiring character elements. It also aimed at verifying the (3) gender differences in self-evaluation of character elements, and (4) academic-year differences in the survey items.

**Methods:**

Medical students’ perceptions were identified through a questionnaire survey among 856 medical students from five South Korean medical schools. The questionnaire comprised items on the achievement level of the character element, importance of character in the medical curriculum, satisfaction with character education in medical schools, and the learning method’s degree of helpfulness. Descriptive statistics, t-test, and one-way ANOVA were used to compare responses.

**Results:**

The importance of eight-character qualities had high average scores, whereas the average scores for satisfaction with character education and achievement level were comparatively low. For the question on each learning method’s helpfulness in gaining the eight-character qualities, the score of team-based learning activities was the highest, followed by club activities, relationships with peers, role modeling of professors, and course study. Regarding satisfaction with character education, male students gave higher scores than female students, manifesting a statistically significant difference. Regarding the importance of the character element in medical education, statistically significant differences existed based on academic year.

**Conclusion:**

Medical students’ perceptions of character education varied according to gender and academic year. They regarded character education highly but were unsatisfied with the current character education at medical schools. Thus, diverse character education curricula must be developed and implemented along with extra-curricular character programs. An effective approach to implementing character education can be discovered by verifying the differences in students’ perceptions based on the character education courses in medical schools.

**Supplementary Information:**

The online version contains supplementary material available at 10.1186/s12909-022-03342-6.

## Background

There is an old saying in Korea that medical practice is an art of compassion (*insul,* 仁術). *Insul* means “benevolent skills that save people’s lives” [1], a definition that implies that doctors must not only have medical knowledge but also possess character qualities [2]. In other words, because medicine is an academic discipline concerned with people’s lives, the field of medical education values character education as highly as advanced medical techniques [3].

Even though there are various views on the concept of character, there is no English expression that perfectly matches the word used in Korean culture. The closest expression in English would be the moral character (”insung”, human nature) [4, 5], Insung refers to the virtues, manners, and conscience that humans can have as humans that can be different from animals. Therefore, this concept can be described as a person's personality, thoughts, attitudes, and behavioral traits [1]. According to prior research, “the character that a doctor requires is the basic attitude, values, and mindset that must be present to perform his or her duties” [6]. This is similar to medical professionalism [7–10], which includes medical knowledge and skills, and in some respects personality can be viewed as a part of medical professionalism. Moral character (hereafter character) is similar to medical professionalism including medical knowledge and skills. However, moral character education is more closely related to the concept of humanism. The Arnold P. Gold Foundation describes humanism in healthcare as a respectful and compassionate relationship between physicians, as well as all other members of the healthcare team, and their patients [11]. A doctor’s identity, morality, and character are described in the Hippocratic Oath [12]. Today, the medical doctor profession requires a strong sense of ethics and character development [5, 13]. This is because doctors exclusively possess expert knowledge and handle the life and well-being of humans directly [5, 13]. In the meantime, medical education in Korea has helped to intensively educate medical professionals, along with the medical knowledge and communication skills necessary to become a good doctor. However, education in the field of attitude is still difficult and seems to be lacking. Therefore, this study focuses on character education in the medical schools of South Korea. In South Korea, after the separation of dispensary from medical practice in 2000, the issue of “how to conduct medical education” came to surface, and the Korean Society of Medical Education began to deliberate on the content and courses to teach medical students what they require in terms of virtues, qualifications, and character qualities under the banner of developing medical professionalism [14]. The Korean Council of Deans of Medical Colleges conducted a study on the future roles and virtues of Korean doctors and identified the Korean doctors’ role: The study classified Korean doctors’ competencies and duties in six domains from an ethical perspective, emphasizing the value of virtues essential for doctors [15]. Korean medical schools reflect the expectations and demands of today’s society and include the desirable virtues and roles in their educational philosophy and goals [16]. In addition, character education is highlighted and developed by multiple medical schools [17]. In 2016, the Korean government passed the Character Education Promotion Law concerning a five-year master plan for character education and announced an organized long-term plan for character education in colleges [18]. A series of such proceedings indicates that the importance of character education is increasing in the ever-changing society of the twenty-first century. This change stemmed from the realization that a “dehumanization of the medical practice” was brought on by conventional medical education, which focused on diverse problems within medical schools and relied on biomedical models for understanding, treating, and managing diseases [19, 20].

Thus far, due to its focus on transferring knowledge and skills, medical education has relatively overlooked curricula in the areas of *insul* and character. Though related curricula have been developed and emphasized with a focus on medical professionalism, humanities, and social medicine over the past decade, it is still difficult to claim that great success or improvement has been achieved. According to a recently published study [21], character-related curricula have been developed and students are learning the material; however, instruction still focuses on medical knowledge and skills, and teaching methods still mainly consist of lectures. As a result, the effectiveness of the education is miniscule [21].

Regarding global trends, a 1999 survey found that 89 medical schools (89%) across 13 Asian countries, Australia, and New Zealand were teaching ethics as a subject [22]. In US and Canadian medical schools, approximately 20 courses were offered (1998–1999), and 71 medical schools (78%) among a total of 141 offered more ethics subjects as of 2000 [23, 24]. This indicates a growing interest in providing education as part of the official curriculum that increases sensitivity to medical ethics and improves ethical decision-making.

As the field of medicine becomes highly diversified and medical technology develops rapidly, it is expected that medical services in the future will differ from those of today, and the doctor-patient relationship will also change. In addition, it is predicted that character education that enhances the understanding of humans and medicine will be even more emphasized so that holistic medical treatment can be available in a demographically diversified and globalized society [25].

Notwithstanding the necessity of character education, very few studies have examined character education’s requirements, such as learners’ attributes, levels, and perception [26]. Although previous literature has analyzed general college students’ spectrum of perception and development of character education [27] as well as character program development [28], studies that targeted medical schools are very rare. In order to develop a character education program for medical school students who are in need for the same more than any other profession, medical schools need to get a comprehensive understanding of needs in order to set educational goals, select educational content, and explore effective educational methods.

We first conducted a study on 'Definition of character for medical education based on expert opinions in Korea' [29], in which we employed a qualitative analysis method through Delphi to identify eight elements of character that must be cultivated in medical students. As an extension of this series of studies, this study intended to investigate medical students' perceptions of the eight character elements revealed by previous studies.

Thus, this study aims to establish a foundation for character education programs in medical schools by the following research objectives; (1) conducting a survey among medical students on the self-evaluation of one’s character based on eight qualities(service and sacrifice, empathy and communication, care and respect, honesty and humility, responsibility and calling, collaboration and magnanimity, creativity and positivity, patience and leadership), the perceived importance of character, and satisfaction with character education at medical school; (2) analysis on the usefulness of learning methods for acquiring character elements; (3) verifying the gender differences in self-evaluation of character elements, and (4) academic-year differences in the survey items.

## Methods

### Research participants

To examine medical students’ perceptions of the eight-character qualities, we conducted an offline paper and pencil questionnaire survey from September to December 2019 among 856 students enrolled in five medical schools in South Korea. We excluded incomplete responses, and finally used data from 728 responses for analysis. Table 1 presents the distribution of the participants’ academic years and gender.

**Table 1:** Academic-year and gender-based distribution of the participants

Gender	Male		Female		Total	
Academic Year	n	%	n	%	n	%
1st year	87	71.3	35	28.7	122	100
2nd year	50	62.5	30	37.5	80	100
3rd year	184	61.3	116	38.7	300	100
4th year	63	54.8	52	45.2	115	100
5th year	51	65.4	27	34.6	78	100
6th year	27	81.8	6	18.2	33	100
Total	462	63.5	266	36.5	728	100

### Survey instruments

To examine medical students’ perception of character, we created a questionnaire survey that consisted of items on the self-assessment of the character elements, importance of character in the medical curriculum, and satisfaction with character education in the medical schools’ curriculum. Responses were scored on a five-point scale (1 = strongly disagree, 2 = disagree, 3 = neither agree nor disagree, 4 = agree, and 5 = strongly agree). The questionnaire also included questions about the degree of helpfulness of the learning methods. Regarding approaches to learning the character qualities, we selected the following categories: club activities (typical extra-curricular activities for medical students during medical school), course studies, role modeling of professors, team-based learning activities, and relationships with peer students. The degree of helpfulness was also scored on a six-point scale (0 = not a helpful method, 1 = strongly disagree. 2 = disagree, 3 = neither agree nor disagree, 4 = agree, 5 = strongly agree). Moreover, we asked respondents an open-ended question on the method of learning each character quality. The content of the questionnaire was reviewed by medical humanities professors and medical education experts to secure content validity.

The reliability of the student response data for self-level, importance, and educational satisfaction of the eight character elements were 0.827, 0.871, and 0.926, respectively. The reliability of the response for each method was 0.955.

### Data method

To analyze the collected data, we used SPSS version 22.0 for Windows (SPSS Inc., Chicago, USA) and conducted frequency analysis, descriptive statistics analysis, *t*-test, and one-way analysis of variance (ANOVA). This study was approved by the Institutional Review Board of Hallym University Industry Foundation (IRB approval no. HIRB-2018–049-1-C).

## Results

### Self-assessment of the eight-character qualities, importance of character in medical education, and satisfaction with character education

**Fig. 1:**
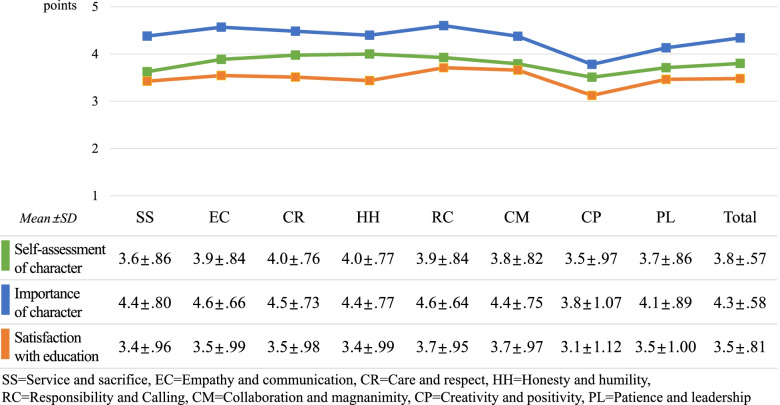
Self-assessment of the eight character qualities, importance of character, and satisfaction with character education

The survey findings on medical students’ self-assessment of the eight-character qualities, importance of character in medical education, and satisfaction with character education are shown in Fig. 1. Regarding their importance, the eight character qualities presented high average scores (4.3 ± 0.58), whereas the average scores for satisfaction with character education (3.5 ± 0.81) and self-assessment level (3.8 ± 0.57) were comparatively low.

In the self-assessment of the eight character qualities, honesty and humility (4.0 ± 0.77) scored the highest. In the importance of character in the medical curriculum, responsibility and calling (4.6 ± 0.64) scored the highest. In terms of satisfaction with education of character qualities, responsibility and calling (3.7 ± 0.95) scored the highest. Creativity and positivity scored the lowest in all three items.

### Analysis on the usefulness of learning methods for acquiring character variables

The survey findings on how helpful each approach is to learn the eight character qualities during medical school studies are shown in Table 2. Team-based learning activities (3.6 ± 0.93) scored the highest, followed by club activities (3.6 ± 1.02), relationships with peer students (3.6 ± 0.87), role modeling of professors (3.4 ± 1.10), and course study (3.0 ± 1.10).

**Table 2:** Degree of helpfulness of the method of learning character qualities (*n* = 728)

Character qualities	Club activities	Course study	Role modeling of professors	Team-based learning activities	Relationships with peer students
Service and sacrifice	3.5 ± 1.39	2.8 ± 1.36	3.3 ± 1.34	3.5 ± 1.25	3.4 ± 1.24
Empathy and communication	3.9 ± 1.17	2.9 ± 1.33	3.4 ± 1.28	3.8 ± 1.10	4.0 ± .97
Care and respect	3.8 ± 1.17	3.0 ± 1.32	3.4 ± 1.25	3.8 ± 1.16	3.9 ± .99
Honesty and humility	3.0 ± 1.35	2.9 ± 1.35	3.4 ± 1.32	3.3 ± 1.21	3.5 ± 1.18
Responsibility and calling	3.7 ± 1.29	3.3 ± 1.29	3.7 ± 1.22	3.8 ± 1.20	3.5 ± 1.22
Collaboration and magnanimity	4.0 ± 1.17	3.1 ± 1.32	3.3 ± 1.33	3.9 ± 1.13	3.8 ± 1.04
Creativity and positivity	3.2 ± 1.43	2.8 ± 1.37	3.1 ± 1.41	3.3 ± 1.26	3.1 ± 1.32
Patience and leadership	3.7 ± 1.22	3.1 ± 1.32	3.5 ± 1.27	3.8 ± 1.12	3.6 ± 1.14
TOTAL	**3.6 ± 1.02**	**3.0 ± 1.10**	**3.4 ± 1.10**	**3.6 ± .93**	**3.6 ± .87**

In learning the qualities of collaboration and magnanimity, empathy, and communication, the respondents mentioned the helpfulness of club activities, team-based learning activities, and relationships with peer students the most. Moreover, they reported that course study and role modeling of professors have been most helpful in learning the qualities of responsibility and calling.

For the open-ended question on the method of learning each character quality during medical school studies, volunteer service activities were mentioned the most, and respondents reported to have learned service and sacrifice, care and respect, empathy and communication, honesty and humility, and responsibility and calling through such activities. YouTube videos were also mentioned as a way of learning creativity and positivity. Other responses include project-based learning, overseas trips, lectures by guest speakers, school festivals, school board or club leadership, and student body activities.

### Gender-based differences in self-assessment of character, importance of character in medical education, satisfaction with character education, and helpfulness of each learning method

We conducted an independent sample *t*-test to examine the differences between male and female students in their current character qualities. The results showed a statistically significant difference in creativity and positivity (*t* = 4.146, *p* < 0.001) as well as patience and leadership (*t* = 2.645, *p* < 0.05). Regarding these two character qualities, male students scored higher than female students.

Regarding the importance of character in medical education, female students (4.7 ± 0.59) showed higher level of perception than male students (4.6 ± 0.66). For responsibility and calling, a statistically significant difference was noted (*t* = -2.291, *p* < 0.01).

Regarding satisfaction with character education in the medical school curriculum, male students (3.5 ± 0.85) scored higher than female students (3.4 ± 0.74), showing a statistically significant difference (*t* = 2.075, *p* < 0.05). In terms of each character quality, gender-based differences were statistically significant in empathy and communication, honesty and humility, creativity and positivity, as well as patience, and leadership.

Upon analyzing the gender difference in the helpfulness of each learning method for character quality, for learning through relationships with peer students, a statistically significant difference (*t* = 2.910, *p* < 0.01) was found between male students (3.7 ± 0.84) and female students (3.5 ± 0.90), as the former scored higher on helpfulness. In terms of each character quality, gender-based differences were statistically significant in service and sacrifice, honesty and humility, responsibility and calling, creativity and positivity, patience, and leadership.

### Academic-year differences in self-assessment of character, importance of character, satisfaction with character education, and the degree of helpfulness for each character learning method

**Fig. 2:**
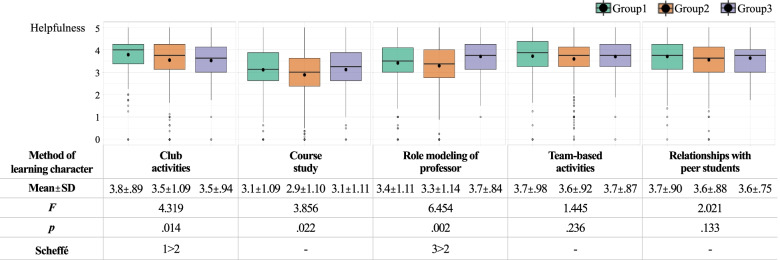
Academic-year differences in helpfulness of the learning method for character qualities (*n* = 728)

To examine the differences in the self-assessed character level, importance of character, satisfaction with character education, and degree of helpfulness of the learning method based on academic year, we divided the students into group 1 (1st and 2nd year), group 2 (3rd and 4th year), and group 3 (5th and 6th year). Results of the one-way ANOVA are shown in Tables 3 and 4 and Fig. 2.

**Table 3:** Academic-year differences in self-assessment and importance of character and satisfaction with character education (*n* = 728)

Character qualities	Self-assessment				Importance					Satisfaction				
	**Group1 (*n* = 202)**	**Group2 (*n* = 415)**	**Group3 (*n* = 111)**	***F***	**Group1 (*n* = 202)**	**Group2 (*n* = 415)**	**Group3 (*n* = 111)**	***F***	**Scheffé**	**Group1 (*n* = 202)**	**Group2 (*n* = 415)**	**Group3 (*n* = 111)**	***F***	**Scheffé**
**Service and sacrifice**	3.6 ± .84	3.6 ± .85	3.6 ± .92	.109	4.5 ± .75	4.4 ± .78	4.2 ± .90	6.65**	1, 2 > 3	3.4 ± 1.01	3.4 ± .94	3.5 ± .96	.854	-
**Empathy and communication**	4.0 ± .79	3.9 ± .86	3.8 ± .87	2.941	4.7 ± .61	4.6 ± .67	4.4 ± .70	6.757**	1, 2 > 3	3.6 ± .99	3.5 ± .98	3.7 ± .99	4.018*	3 > 2
**Care and respect**	4.0 ± .78	4.0 ± .76	4.0 ± .76	.024	4.6 ± .66	4.5 ± .73	4.3 ± .79	6.534**	1 > 3	3.6 ± 1.00	3.5 ± .97	3.6 ± 1.01	1.679	-
**Honesty and humility**	4.8 ± .76	4.0 ± .79	4.0 ± .68	1.388	4.5 ± .76	4.4 ± .73	4.1 ± .87	11.429***	1, 2 > 3	3.5 ± 1.03	3.4 ± .97	3.6 ± .98	2.863	-
**Responsibility and calling**	3.9 ± .87	3.9 ± .85	4.0 ± .71	.671	4.7 ± .60	4.6 ± .64	4.4 ± .66	5.637**	1 > 3	3.8 ± .97	3.7 ± .93	3.7 ± .98	.724	-
**Collaboration and magnanimity**	3.8 ± .86	3.8 ± .81	3.8 ± .81	.260	4.4 ± .77	4.4 ± .74	4.3 ± .74	.366	-	3.8 ± 1.05	3.6 ± .93	3.7 ± .98	2.454	-
**Creativity and positivity**	3.6 ± .96	3.5 ± .98	3.4 ± .94	.655	3.9 ± 1.03	3.8 ± 1.08	3.7 ± 1.09	1.284	-	3.2 ± 1.12	3.0 ± 1.09	3.3 ± 1.19	3.022*	-
**Patience and Leadership**	3.8 ± .82	3.7 ± .87	3.7 ± .87	.711	4.2 ± .87	4.1 ± .89	4.0 ± .91	2.764	-	3.5 ± 1.00	3.4 ± 1.00	3.7 ± .95	4.247*	3 > 2
**TOTAL**	3.8 ± .53	3.8 ± .59	3.8 ± .55	.659	4.4 ± .57	4.3 ± .56	4.2 ± .62	7.555**	1, 2 > 3	3.6 ± .84	3.4 ± .77	3.6 ± .88	3.030*	-

^*^p < .05, **p < .01, ***p < .001

**Table 4:** Academic-year differences in the helpfulness of the learning method for the eight character qualities (*n* = 728)

Category		Service and sacrifice	Empathy and communication	Care and respect	Honesty and humility	Responsibility and calling	Collaboration and magnanimity	Creativity and positivity	Patience and leadership
**Club activities**	**Group1 (*n* = 202)**	3.5 ± 1.38	4.3 ± .96	4.1 ± 1.03	3.1 ± 1.33	3.9 ± 1.30	4.3 ± 0.97	3.3 ± 1.42	3.8 ± 1.16
	**Group2 (*n* = 415)**	3.5 ± 1.41	3.8 ± 1.25	3.7 ± 1.26	3.0 ± 1.39	3.7 ± 1.30	3.9 ± 1.25	3.1 ± 1.47	3.7 ± 1.29
	**Group3 (*n* = 111)**	3.3 ± 1.31	3.8 ± 1.11	3.7 ± 0.99	3.1 ± 1.20	3.5 ± 1.20	3.7 ± 1.07	3.4 ± 1.25	3.6 ± 1.05
	***F***	1.096	10.763***	7.219**	1.549	2.692	11.594***	2.829	1.121
	**Scheffé**		1 > 2,3	1 > 2,3			1 > 2,3		
**Course studies**	**Group1 (*n* = 202)**	2.8 ± 1.37	3.1 ± 1.32	3.1 ± 1.33	3.0 ± 1.31	3.4 ± 1.25	3.3 ± 1.32	3.0 ± 1.38	3.3 ± 1.29
	**Group2 (*n* = 415)**	2.7 ± 1.33	2.8 ± 1.34	2.8 ± 1.32	2.8 ± 1.38	3.3 ± 1.31	3.0 ± 1.34	2.6 ± 1.37	3.0 ± 1.32
	**Group3 (*n* = 111)**	2.9 ± 1.44	3.1 ± 1.29	3.2 ± 1.23	3.1 ± 1.27	3.3 ± 1.31	3.2 ± 1.22	2.9 ± 1.31	3.2 ± 1.34
	***F***	.773	4.207*	5.444**	3.216*	.646	2.855	4.317*	3.918*
	**Scheffé**		1 > 2	1,3 > 2				1 > 2	1 > 2
**Role modeling of professors**	**Group1 (*n* = 202)**	3.3 ± 1.37	3.4 ± 1.26	3.5 ± 1.25	3.4 ± 1.32	3.7 ± 1.27	3.2 ± 1.36	3.1 ± 1.37	3.6 ± 1.20
	**Group2 (*n* = 415)**	3.2 ± 1.38	3.2 ± 1.33	3.3 ± 1.29	3.3 ± 1.36	3.7 ± 1.26	3.2 ± 1.36	3.0 ± 1.46	3.4 ± 1.31
	**Group3 (*n* = 111)**	3.8 ± 1.07	3.7 ± 1.03	3.8 ± 0.99	3.7 ± 1.05	4.0 ± 0.88	3.7 ± 1.05	3.3 ± 1.28	3.7 ± 1.22
	***F***	6.996**	6.364**	8.979***	4.800**	2.605	7.359**	1.922	2.239
	**Scheffé**	3 > 1,2	3 > 2	3 > 2	3 > 2		3 > 1,2		
**Team-based learning activities**	**Group1 (*n* = 202)**	3.5 ± 1.35	4.0 ± 1.07	3.9 ± 1.24	3.3 ± 1.27	3.8 ± 1.28	4.0 ± 1.12	3.4 ± 1.30	3.8 ± 1.24
	**Group2 (*n* = 415)**	3.5 ± 1.24	3.8 ± 1.11	3.8 ± 1.12	3.3 ± 1.19	3.7 ± 1.17	3.8 ± 1.13	3.2 ± 1.26	3.7 ± 1.10
	**Group3 (*n* = 111)**	3.5 ± 1.12	3.8 ± 1.06	3.8 ± 1.16	3.5 ± 1.15	3.9 ± 1.13	3.9 ± 1.15	3.4 ± 1.13	3.8 ± 1.00
	***F***	.023	3.099*	.664	1.150	1.133	2.424	2.720	.983
	**Scheffé**		1 > 2						
**Relationships with peer students**	**Group1 (*n* = 202)**	3.5 ± 1.25	4.2 ± 0.94	4.1 ± 0.95	3.6 ± 1.30	3.6 ± 1.34	3.9 ± 1.08	3.1 ± 1.35	3.6 ± 1.19
	**Group2 (*n* = 415)**	3.3 ± 1.28	3.9 ± 0.99	3.9 ± 1.01	3.5 ± 1.17	3.5 ± 1.17	3.8 ± 1.05	3.0 ± 1.33	3.6 ± 1.16
	**Group3 (*n* = 111)**	3.5 ± 1.04	3.9 ± 0.96	3.8 ± 0.95	3.6 ± 0.95	3.6 ± 1.19	3.8 ± 0.95	3.3 ± 1.16	3.6 ± 1.00
	***F***	1.857	5.054**	6.246**	1.212	.221	1.540	1.905	.320
	**Scheffé**		1 > 2,3	1 > 2,3					

^*^p < .05, **p < .01, ***p < .001

Regarding academic-year differences in self-assessment of the eight character qualities, students showed a tendency to score themselves lower as they went up in academic year. However, this difference was not statistically significant.

For the importance of the character element in medical education, academic-year differences were statistically significant (*F* = 7.555, *p* < 0.01) for the following character qualities: service and sacrifice, empathy and communication, care and respect, honesty and humility, and responsibility and calling. As students moved up in the academic year, the level of the perceived importance of character in medical education decreased. Results of the Scheffé post-test showed that group 1 and group 2 perceived the importance of character element more highly than did group 3.

Concerning satisfaction with character education, group 2 marked it low (3.4 ± 0.77 points), but clinical medicine students showed higher satisfaction level (3.6 ± 0.88 points) than did group 1 (3.6 ± 0.84 points). There was a statistically significant academic-year difference in the following character qualities: empathy and communication, creativity and positivity, and patience and leadership. Analysis of the post-test results revealed that group 3 showed higher level of satisfaction with character education for empathy and communication as well as patience and leadership than did group 2.

We compared the student groups by academic year in terms of the helpfulness of the five different approaches to learning character qualities. The results showed a statistically significant difference in club activities (*F* = 4.319, *p* < 0.05) and role modeling of professors (*F* = 6.454, *p* < 0.01). The post-test results showed that club activities were reported to be more helpful to group 1 than group 2, and role modeling of professors was reported to be more helpful to group 3 than group 2. In terms of each character quality, a statistically significant academic-year difference existed in the five approaches to learning empathy and communication. Group 1 found club activities most helpful, whereas group 2 and group 3 found relationships with peer students most helpful for learning character qualities.

For learning character qualities through club activities, the academic-year difference was statistically significant for empathy and communication (*F* = 10.763, *p* < 0.001), care and respect (*F* = 7.219, *p* < 0.01), and collaboration and magnanimity (*F* = 11.594, *p* > 0.001). Club activities were reported to be more helpful for learning care and respect as well as collaboration and magnanimity among group 1 than group 2 or group 3. The post-test results also showed statistically significant differences.

Learning character qualities through course studies scored lower than the other learning approaches. Statistically significant academic-year differences were found in the following character qualities: care and respect (*F* = 5.444, *p* < 0.01), honesty and humility (*F* = 3.216, *p* < 0.05), creativity and positivity (*F* = 4.317, *p* < 0.05), and patience and leadership (*F* = 3.918, *p* < 0.05).

Concerning learning through the role modeling of professors, its degree of perceived helpfulness tended to increase as the students moved up in academic year. The academic-year differences were statistically significant for the following characteristics: service and sacrifice (*F* = 6.996, *p* < 0.01), empathy and communication (*F* = 6.364, *p* < 0.01), care and respect (*F* = 8.979, *p* < 0.001), honesty and humility (F = 4.800, *p* < 0.01), and collaboration and magnanimity (*F* = 7.359, *p* < 0.01).

Regarding learning through team-based learning activities, the academic-year differences were statistically significant in empathy and communication (*F* = 3.099, *p* < 0.05), whereas for learning through relationships with peer students, statistically significant academic-year differences existed for empathy and communication (*F* = 5.054, *p* < 0.01) and care and respect (*F* = 6.246, *p* < 0.01). Learning character qualities through relationships with peer students was reported to be highly helpful for group 2.

## Discussion

In this study, medical students’ self-assessed character level was 3.8 points, which shows a small discrepancy with that of nursing students (3.4 ± 0.57 points) in the health care field in terms of the average character achievement score [30]. Medical students’ perceived importance of character qualities was 4.3 points on average, similar to that of nursing students (4.4 ± 0.44 points) [30].

The medical students gave the importance of each character quality in the following order: 1) responsibility and calling, 2) empathy and communication, 3) care and respect, 4) honesty and humility, 5) service and sacrifice, 6) collaboration and magnanimity, 7) patience and leadership, and 8) creativity and positivity. Similarly, nursing students regarded responsibility as the most important character quality [28]. These findings suggest that medical and nursing students have the shared goal of saving people’s lives by taking the Hippocratic Oath and Nightingale Pledge, respectively.

The assessment of character level showed that male students regarded themselves as having higher levels of character achievement than female students did. In the assessment of character achievement by academic year, no statistically significant difference was found. This finding is in line with a previous study that found no significant academic-year differences in nursing students’ assessment of character achievement [30]. The reason for analyzing the gender difference in this study is that the characteristics of learners in schools are condition variables that should not be overlooked by teachers. Furthermore, it can be expected that if the results of this study are used as basic data for student guidance and student counseling, it will be helpful for teachers to recognize gender difference.

Meanwhile, regarding the learning method for improving character quality, the students reported that course studies and role modeling of professors are the most effective ways to learn the sense of responsibility and calling. They reported that empathy and communication can be learned through relationships with peer students, collaboration and magnanimity can be learned through club activities and team-based learning activities, and the service and sacrifice mindset can be learned through volunteer service activities.

In particular, the role modeling of professors was reported to be helpful for learning all eight character qualities by senior medical students as opposed to junior medical students. This indicates that developing character qualities as medical students through the professors’ examples are more helpful than club activities, team-based learning activities, or relationships with peer students as students move up in academic year.

This study has important implications for establishing the goals and directions for character education and selecting the educational methods. Character qualities can be learned through the official curriculum, but this study’s findings verified that character can be developed through covert education (e.g., role modeling of professors as opposed to course studies) and extra-curricular activities such as volunteer service. Therefore, medical schools need to establish meticulous plans at the time of developing character education programs to establish an atmosphere or culture that considers potential educational opportunities. In addition, it seems to be helpful to guarantee physical time for extra-curricular activities and establish a policy or graduation requirement that encourages extra-curricular activities. Holden et al. provided some useful examples of how to develop activities and assessments according to the domain and subdomain identified as the professional identity formation framework [31].

This study had several limitations. Even though we selected 5 medical schools among 40 medical schools in South Korea, the study findings are too limited to be generalized to all medical schools, considering the regions and school types (national/private). Character education available at each medical school differs in terms of content and format; therefore, it is difficult to apply the findings on the method of learning character qualities to all medical students. Nevertheless, considering the general characteristics of medical students in South Korea, each medical school can use this study’s findings as data for analyzing the students’ demands when developing a character education program.

Some further research can be done based on the results of this study. We could first start with a study closely looking into the concept of character education not just according to its elements but by the definitions given by experts in medical education. The definition of professionalism is quite controversial, and with humanities in healthcare. Through the collected expert opinions, it will be possible to establish a clearer concept and easier to identify this phenomenon at the institutional or academic level. In this regard, a follow-up study was conducted just after this study and is on the verge of formal publication [32]. In addition, these concepts and definitions can lead to a framework of character education. The framework for humanism in healthcare that the Arnold P. Gold Foundation provides [11], including seven core attributes (integrity, excellence, compassion & collaboration, altruism, respect & resilience, empathy, and service), is a good example.

To enhance any kind of curriculum, we must take evaluation as a key element. In Korean education, assessment is taken seriously, and the current Korean health system science, which includes professionalism, humanities, value-based medicine, and so on, is a main component of accreditation for medicals school in Korea [33]. Based on the analysis results of factors with low satisfaction rates in this study as well as on the preferred character education method, a study on how to emphasize the medical humanities curriculum or medical professionalism can also be done. A follow-up study on whether character development was achieved (through pre-and post-evaluation at the time of admission and graduation after curriculum reform) would be desirable. We also need some specific answers to the problems of character education rather than simply defining them.

We can see from these study results that the students do not think that formal course studies help them to learn character qualities; instead; character is currently cultivated through hidden or non-curricular courses and activities. Thus, research on the development of measures that can assess the level of character development intended for medical schools and student evaluation methods would be useful. As an example, morals and character are assessed during recruiting and training in the US military [34]. As such, it is necessary to develop a character scale that can measure character when entering and graduating from medical school.

## Conclusion

While medical students recognize the importance of character qualities, they manifest a low level of satisfaction with the character education available in the medical school curriculum. Among the many learning methods for achieving character qualities, learning through course studies was perceived to be less helpful than team-based learning activities or club activities. There were gender-based and academic-year differences in the self-assessed character achievement level, importance of character, satisfaction with character education, and helpfulness of the learning method.

In conclusion, the medical students’ perceived level of character education differed based on gender and academic year. They deemed character education important but had a low level of satisfaction with the current character education programs. Therefore, based on the findings of demand analysis from this study and by verifying the differences in students’ perceptions based on character education at medical schools, it is suggested that an effective method for character education can be found when developing and implementing a diverse curriculum and extra-curricular character programs.

## Supplementary Information

**Additional file 1.** Questionnaire to identify the character elements of medical students.

## Data Availability

The datasets generated and/or analysed during the current study are not publicly available due privacy and confidentiality reasons but are available from the corresponding author on reasonable request.

## Abbreviations

ANOVA: Analysis of variance
IRB: Institutional Review Board
HIRB: Hallym Institutional Review Board
SS: Service and sacrifice
EC: Empathy and communication
CR: Care and respect
HH: Honesty and humility
RC: Responsibility and calling
CM: Collaboration and magnanimity
CP: Creativity and positivity
PL: Patience and leadership
SD: Standard deviation
