# Supplementary material for: Medical students’ self-evaluation of character, and method of character education
Source: BMC Med Educ. 2022 Apr 13;22:271. doi: 10.1186/s12909-022-03342-6 (PMC9006595; doi:10.1186/s12909-022-03342-6)
Supplement: Supplementary file 1 — Additional file 1. Questionnaire to identify the character elements of medical students. [file 12909_2022_3342_MOESM1_ESM.docx]

**Questionnaire to identify the character elements of medical students**

| This questionnaire is to find out the level of medical students in Korea regarding the 8 character elements. This questionnaire is not a test paper and is not intended to evaluate how well you did or did not do. Therefore, there is no right answer to each question. Just indicate your honest opinion. |
| --- |

※ The following are 8 character elements and sub-elements. For each core element, please indicate your current level, importance in medical education, and satisfaction with education in the appropriate score.

[Response method]

1=strongly disagree. 2=disagree, 3=neither agree nor disagree, 4=agree, 5=strongly agree

| **Element** | **Level** | **Importance** | **Satisfaction** |
| --- | --- | --- | --- |
| **1. Service and sacrifice**  - Service, sacrifice, devotion, altruism, compassion, concession, philanthropy, gratitude | 1----2----3----4----5 | 1----2----3----4----5 | 1----2----3----4----5 |
| **2. Empathy and communication**  -Communication skills, empathy, expression, listening, conflict management | 1----2----3----4----5 | 1----2----3----4----5 | 1----2----3----4----5 |
| **3. Care and respect**  -Communication skills, empathy, expression, listening, conflict management | 1----2----3----4----5 | 1----2----3----4----5 | 1----2----3----4----5 |
| **4. Honesty and humility**  - Honesty, sincerity, humility, morality, sincerity, integrity, ethical judgment | 1----2----3----4----5 | 1----2----3----4----5 | 1----2----3----4----5 |
| **5. Responsibility and calling**  - Responsibility, medical ethics, accountability, sense of calling, sense of duty | 1----2----3----4----5 | 1----2----3----4----5 | 1----2----3----4----5 |
| **6. Collaboration and magnanimity**  - Collaboration, inclusion, sense of community, exchange, interdependence | 1----2----3----4----5 | 1----2----3----4----5 | 1----2----3----4----5 |
| **7. Creativity and positivity**  - Creativity, positivity, openness, courage, insight | 1----2----3----4----5 | 1----2----3----4----5 | 1----2----3----4----5 |
| **8. Patience and leadership**  - Patience, leadership, self-reflection, self-identity, social awareness | 1----2----3----4----5 | 1----2----3----4----5 | 1----2----3----4----5 |

※ For each of the 8 character elements, please indicate how you learned them through your medical school life by putting a ✔ in the appropriate box. Duplicate notation is possible, and if it is indicated in “Other”, please describe it in detail.

[Response method]

0=not a helpful method (no check)

1=strongly disagree. 2=disagree, 3=neither agree nor disagree, 4=agree, 5=strongly agree

| **Element** | **the method of learning character qualities** | | | | | |
| --- | --- | --- | --- | --- | --- | --- |
|  | **Club**  **activities** | **Course study** | **Role modeling of professors** | **Team-based learning activities** | **Relationships with peer students** | **Other** |
| **1. Service and sacrifice** | **①** | **②** | **③** | **④** | **⑤** |  |
| **2. Empathy and communication** | **①** | **②** | **③** | **④** | **⑤** |  |
| **3. Care and respect** | **①** | **②** | **③** | **④** | **⑤** |  |
| **4. Honesty and humility** | **①** | **②** | **③** | **④** | **⑤** |  |
| **5. Responsibility and calling** | **①** | **②** | **③** | **④** | **⑤** |  |
| **6. Collaboration and magnanimity** | **①** | **②** | **③** | **④** | **⑤** |  |
| **7. Creativity and positivity** | **①** | **②** | **③** | **④** | **⑤** |  |
| **8. Patience and leadership** | **①** | **②** | **③** | **④** | **⑤** |  |
